# Zinc Coordination Compounds with Benzimidazole Derivatives: Synthesis, Structure, Antimicrobial Activity and Potential Anticancer Application

**DOI:** 10.3390/ijms23126595

**Published:** 2022-06-13

**Authors:** Anita Raducka, Marcin Świątkowski, Izabela Korona-Głowniak, Barbara Kaproń, Tomasz Plech, Małgorzata Szczesio, Katarzyna Gobis, Małgorzata Iwona Szynkowska-Jóźwik, Agnieszka Czylkowska

**Affiliations:** 1 Institute of General and Ecological Chemistry, Faculty of Chemistry, Lodz University of Technology, Żeromskiego 116, 90-924 Łódź, Poland; marcin.swiatkowski@p.lodz.pl (M.Ś.); malgorzata.szczesio@p.lodz.pl (M.S.); malgorzata.szynkowska@p.lodz.pl (M.I.S.-J.); 2 Department of Pharmaceutical Microbiology, Medical University of Lublin, Chodźki 1, 20-093 Lublin, Poland; iza.glowniak@umlub.pl; 3 Department of Clinical Genetics, Medical University of Lublin, Radziwilłłowska 11, 20-080 Lublin, Poland; barbara.kapron@umlub.pl; 4 Department of Pharmacology, Medical University of Lublin, Radziwiłłowska 11, 20-080 Lublin, Poland; tomasz.plech@umlub.pl; 5 Department of Organic Chemistry, Faculty of Pharmacy, Medical University of Gdansk, Gen. Hallera 107, 80-416 Gdańsk, Poland; katarzyna.gobis@gumed.edu.pl

**Keywords:** metal complexes, coordination compounds, biological activity, biomedical applications, structural studies, metallodrugs, bioorganometallic chemistry, pharmacological activity, lung cancer, glioblastoma, neuroblastoma, adenocarcinoma, benzimidazole derivatives, thermogravimetric analysis, FTIR spectra, ADME analysis, MTT assay, medicinal inorganic chemistry

## Abstract

Developing new, smart drugs with the anticancer activity is crucial, especially for cancers, which cause the highest mortality in humans. In this paper we describe a series of coordination compounds with the element of health, zinc, and bioactive ligands, benzimidazole derivatives. By way of synthesis we have obtained four compounds named **C1**, **C2**, **C4** and **C4.** Analytical analyses (elemental analysis (EA), flame atomic absorption spectrometry (FAAS)), spectroscopic (Fourier transform infrared spectroscopy (FT-IR), mass spectrometry (MS)) and thermogravimetric (TG) methods and the definition of crystal structures were used to explore the nature of bonding and to elucidate the chemical structures. The collected analytical data allowed the determination of the stoichiometry in coordination compounds, thermal stability, crystal structure and way of bonding. The cytotoxicity effect of the new compounds as a potential antitumor agent on the glioblastoma (T98G), neuroblastoma (SK-N-AS) and lung adenocarcinoma (A549) cell lines and human normal skin fibroblasts (CCD-1059Sk) was also determined. Cell viability was determined by the MTT assay. The results obtained confirmed that conversion of ligands into the respective metal complexes significantly improved their anticancer properties. The complexes were screened for antibacterial and antifungal activities. The ADME technique was used to determine the physicochemical and biological properties.

## 1. Introduction

There is no doubt that minerals are necessary for the proper development of plant and animal organisms. One of the necessary microelements is zinc, isolated as a pure metal in 1746 in Germany by Andreas Marggraf [1]. Zinc is commonly known as the element of health. Zinc is supplied to the body mainly with food of plant and animal origin, to a lesser extent through the respiratory system and skin, and excreted with feces and partly with urine [2]. Zinc absorption occurs in the small intestine, large intestine and stomach, and its absorption depends on environmental pollution, stress, poor diet, exercise, age, excess sugar, low protein and high iron intake and high doses of phytates derived from plant products [3,4,5,6,7,8]. Zinc is a necessary microelement for regular functioning of plant and animal organisms. Low and high concentrations can cause many diseases. Zinc deficiency can inhibit the growth of children as it is involved in bone metabolism [7,9]. It has an effect on the immune system and inhibits inflammation, and its deficiency has been found in children with pneumonia and bronchitis [10,11]. The results of a too low level of zinc are changes in the hormone balance, an increase in glucose tolerance [12], exacerbation of symptoms in depression [13], potency disorders [14] and disturbed nerve conduction [15]. The proper level of this element determines the activity of antioxidant enzymes, including zinc–copper superoxide dismutase, and it is thanks to its presence that damage to purine bases in DNA, which is considered one of the causes of cancer, is avoided [16,17,18]. A higher Cu/Zn ratio contributes to the development of non-small-cell lung cancer [19]. Due to the aforementioned properties of zinc, it was selected for syntheses with medically active ligands such as benzimidazoles. The therapeutic potential of a benzimidazole molecule dates back to 1944 [20]. The presence of the benzimidazole molecule in bioactive compounds has an impact on antiparasitic, anticonvulsant, analgesic, antihistaminic, antiulcer, antihypertensive, antiviral, anticancer, antifungal and anti-inflammatory agents, and this compound has anticoagulant properties [21,22,23,24,25,26,27,28,29,30]. In this work, we focused on the design and creation of medically active coordination compounds containing a benzimidazole core, with potential anticancer properties and an element essential for all living organisms—zinc [31,32,33,34,35,36,37,38,39,40]. Currently, the leading cause of cancer-related mortality worldwide is lung cancer. Each year, about 1.82 million people are diagnosed with lung cancer, and about 85% of these people die from the disease [41]. There are two classes of lung cancer, approximately 85% of which are non-small-cell lung cancer. Additionally, there are two histopathological subtypes in this class, and lung adenocarcinoma (LADC) is more common [42]. Another cancer that causes high mortality is neuroblastoma (NBL). It is one of the most common extracranial solid tumors. It is a tumor originating in the cells of the nerve crests, which normally form the sympathetic ganglia and the adrenal medulla. NBL accounts for about 8–10% of all malignant neoplasms in the group of children and adolescents [41,43]. Coordination compounds of various benzimidazole derivatives with selected dn-electron metals give promising results in the search for new, better and more selective anticancer drugs. For the synthesis of complexes with these N-donor ligands, mainly metal ions such as ruthenium, platinum, palladium and silver were used [44,45,46,47,48,49,50]. In addition, very good research results have been achieved for complexes in which the central ions are biometals necessary for the proper functioning of the human body [51,52]. In all cases [44,45,46,47,48,49,50,51,52], the authors describe the anticancer properties of the synthesized complexes. Additionally, the interaction of the obtained compounds with DNA has been investigated [44,45,46,48,51]. The biological studies described in [44,45,46,47,48,49,50,51,52] show that the metal–biologically active ligand research direction gives hope for obtaining new anticancer drugs. These papers clearly show not only that the organic ligand plays an important role, but also that the selection of the metal ion has a great influence on the physicochemical and biological properties of the synthesized coordination compounds. The electronic structure of the metal ion, the number of available coordination sites in the organic ligand, the type of bond or interaction between the metal ion and the ligand and the coordination number of the complex give the biological properties of the studied coordination compounds. The synthesis and characterization of an increasing number of coordination compounds show the role of transition metal complexes in medicine.

The presented paper is the continuation of our work on the synthesis and characterization of metal (II) complexes with benzimidazole derivatives [52]. Now, we describe eight novel solid coordination compounds with not commercially available organic ligands. The benzimidazole derivatives were designed using a structure-based drug design approach. All synthesized compounds were characterized by F-AAS, FTIR, TGA and XRD analysis. Moreover, for all coordination compounds, in vitro cytotoxicity for glioblastoma (T98G), neuroblastoma (SK-N-AS) and lung adenocarcinoma (A549) cell line inhibition and antibacterial and antifungal activities were measured.

## 2. Results and Discussion

### 2.1. Synthesis

#### 2.1.1. Ligand Synthesis

**Scheme 1:**
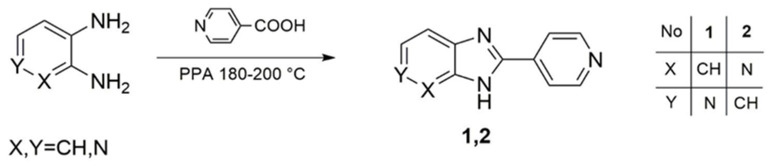
Synthesis of ligand 1 (**L1**) and 2 (**L2**).

**Scheme 2:**
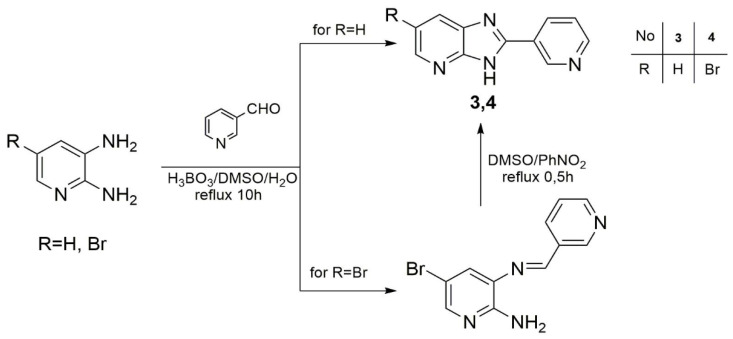
Synthesis of ligands 3 (**L3**) and 4 (**L4**).

Ligands **L1** and **L2** were synthesized by the condensation and cyclization of 2,3-diaminopyridine or 3,4-diaminopyridine with isonicotinic acid in PPA (Scheme 1), and ligands **L3** and **L4** were obtained by reacting 2,3-diaminopyridine or 5-bromo-2,3-diaminopyridine with 3-pyridinecarboxaldehyde in the presence of boric acid in a mixture of water and DMSO (Scheme 2). These syntheses we have previously described [52].

#### 2.1.2. Complex Synthesis

**Scheme 3:**
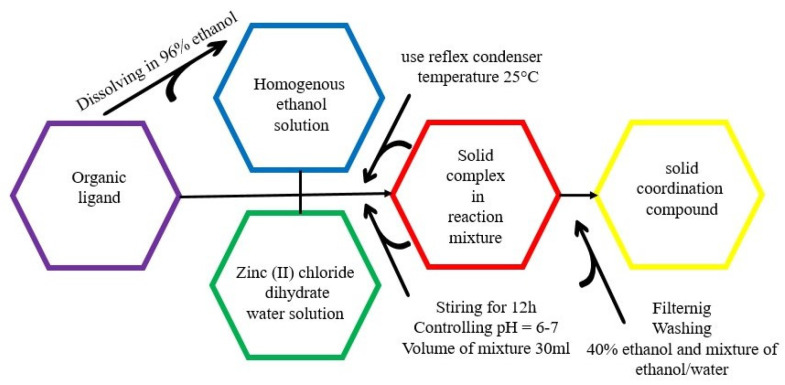
Synthesis of solid zinc (II) coordination compounds.

All complexes **C1**, **C2**, **C3** and **C4** were obtained using the method described in an earlier article [52]. Benzimidazole derivatives (0.25 mmol) and zinc chloride dihydrates (0.25 mmol) were dissolved in 96% *v*/*v* ethanol until homogeneous solutions were obtained. Using a reflux condenser and a magnetic stirrer, they were mixed for 12 h. The total volume of the reaction mixture was 30 mL. The reaction was carried out at constant room temperature (25 °C) and controlled pH (6–7) until the formation of a precipitate of coordination compounds, which was then washed with 40% EtOH and a mixture of EtOH and H_2_O (vol = 1/1). The reaction products were air-dried at room temperature (Scheme 3). The obtained coordination compounds were defined by means of elemental C/H/N analysis and determination of Zn (II) content (Table 1), FTIR spectra and TG-MS technique, as well as by solving the crystalline structures of the obtained single crystals.

### 2.2. MTT Cytotoxicity Assay

Ligands alone (**L1**, **L2**, **L3** and **L4**) and their Zn (II) metal complexes were screened for anticancer activity against glioblastoma, neuroblastoma and lung carcinoma cell lines (T98G, SK-N-AS and A549, respectively). Moreover, in order to assess the selectivity of the anticancer effect, the investigated compounds were also evaluated against human normal skin fibroblasts (CCD-1059Sk). Etoposide, which belongs to topoisomerase IIα inhibitors, was used as a reference drug. The results obtained confirmed that the conversion of ligands into the respective metal complexes significantly improved their anticancer properties (Table 2). All of them showed much stronger activity than etoposide, which is a component of therapy for glioblastoma, neuroblastoma and lung cancer. Unfortunately, the increased cytotoxicity of the metal complexes was also observed in relation to normal cells. Only **C1** and **C4** complexes turned out to selectively inhibit the growth of T98G glioblastoma cells with IC50 values equaling 30.05 and 24.29 µg/mL, respectively. Although most of the investigated metal complexes cannot be used as a potential systemic treatment for cancer due to their cytotoxic effect on normal cells, they can be integrated into different drug delivery systems. Such carriers are able to precisely deliver anticancer drugs into the tumor sites, especially those located in the brain, which is prevented by the blood–brain barrier (BBB) against toxic xenobiotics carried in the bloodstream.

### 2.3. Antimicrobial Activity

The antimicrobial activity of Zn complexes, free ligands and reference drugs was tested against six strains of Gram-positive bacteria, five strains of Gram-negative rods and three strains of yeasts. Antimicrobial properties were expressed as minimum inhibitory concentration (MIC) in milligrams per liter (Table 3). The antimicrobial activity of Zn complexes was compared with that of the antimicrobial and antifungal properties of the appropriate ligands. Vancomycin (Van), ciprofloxacin (Cip) and nystatin (Nys) were used as the standard drugs. The tested compounds showed no bioactivity against Gram-negative bacteria and yeasts. Regarding Gram-positive bacteria, mild bioactivity was detected, with a slight increase for Zn complexes.

### 2.4. FTIR Spectra

**Figure 1:**
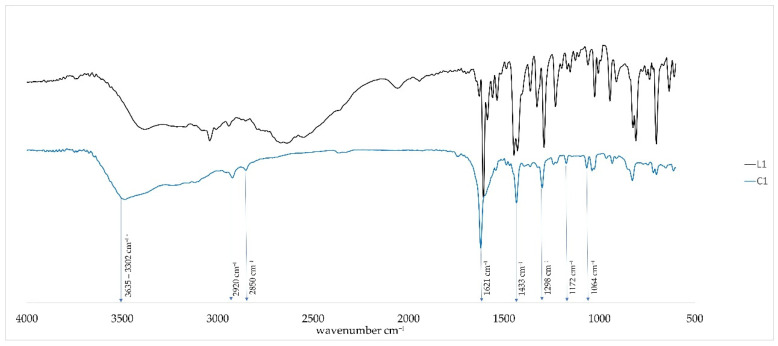
FTIR spectrum of free organic ligands **L1** and complex **C1**.

**Figure 2:**
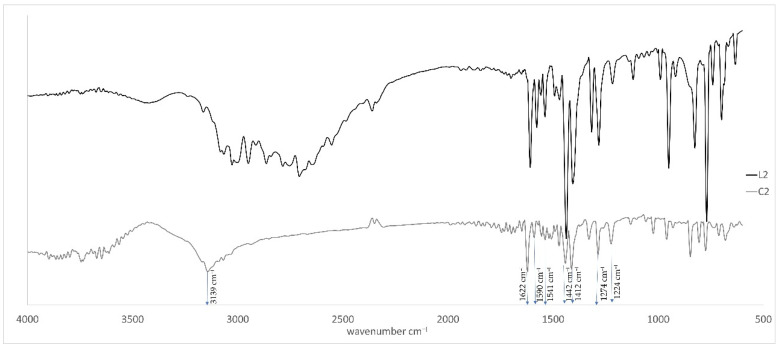
FTIR spectrum of free organic ligands **L2** and complex **C2**.

**Figure 3:**
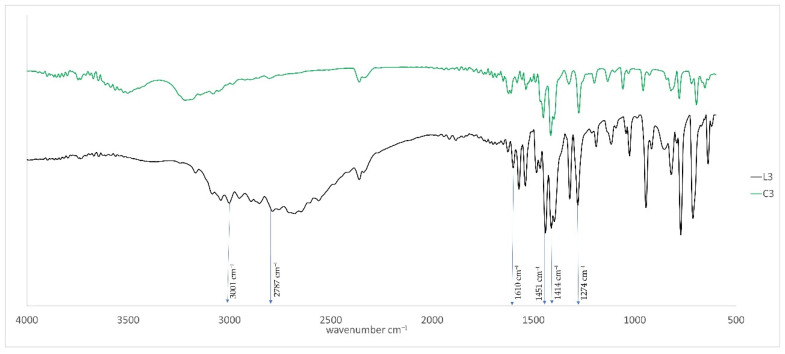
FTIR spectrum of free organic ligands **L3** and complex **C3**.

**Figure 4:**
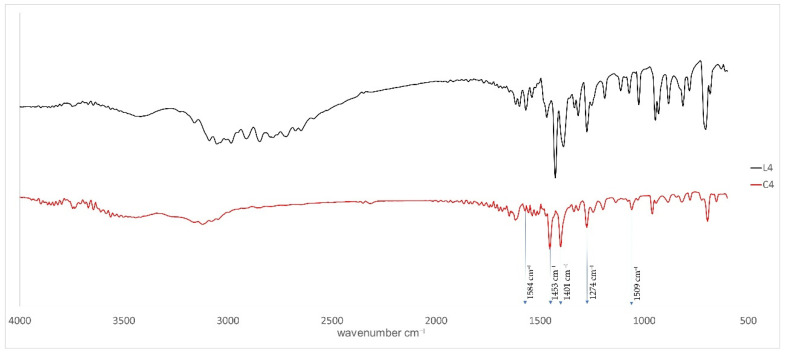
FTIR spectrum of free organic ligands **L4** and complex **C4**.

The infrared spectra of free ligands with the corresponding zinc coordination compounds were collected within the range 4000–500 cm^−1^ and are shown in Figure 1, Figure 2, Figure 3 and Figure 4. In the case of the coordination compound **C1**, we can observe a broad absorption band at 3635–3302 cm^−1^ which is assigned to the stretching vibrations ν(OH) of water molecules. In the IR spectra of complexes, the ligand bands are retained but undergo considerable shifts to a higher frequency. The greatest displacement in the spectra of compounds **C3** and **C4** occurs for the bands corresponding to imidazole ring vibrations, which attests to coordination through the pyridine nitrogen atom. In general, it can be observed that the spectra of the coordination compounds are changed in comparison with the spectra of the free ligands. Some peaks fade away, while others shift towards higher or lower wavelengths. The fundamental ν(NH) and ν(CH) vibrational stretching modes in the free ligands (from the imidazole ring) occur in the range 3500–2543 cm^−1^ and are shifted towards higher wavenumbers (3600–2718 cm^−1^) as a result of coordination. For free benzimidazole ligand derivatives, vibration modes of ν(C=N) and ν(C=C) are visible in the ranges 1609–1539 cm^−1^ and 1447–1411 cm^−1^, and for complexes, these frequencies shift towards higher or lower frequencies with peaks of 1621 and 1433 cm^−1^ for **C1**; 1622, 1590, 1541, 1442 and 1412 cm^−1^ for **C2**; 1610, 1451 and 1414 cm^−1^ for **C3**; and 1584, 1619, 1538 and 1453 cm^−1^ for **C4**. In the discussed spectra of both ligands and complexes, we can observe β(CH) and γ(CH) mode vibrations in the range of 1274–1024 cm^−1^. All these changes are results of coordination between the metal ions and organic ligand. These changes indicate metal–nitrogen bonds.

### 2.5. Thermal Study

**Figure 5:**
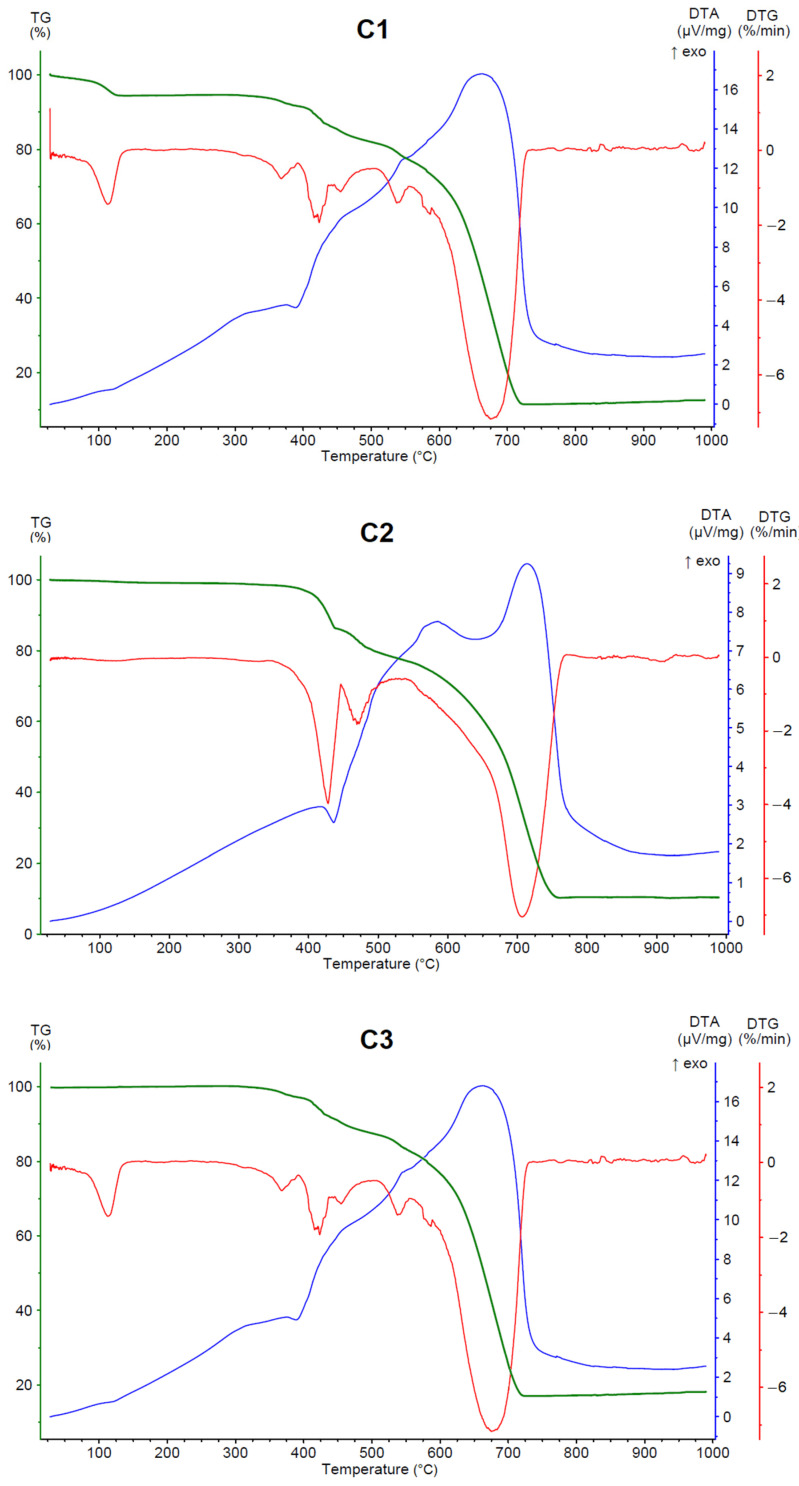

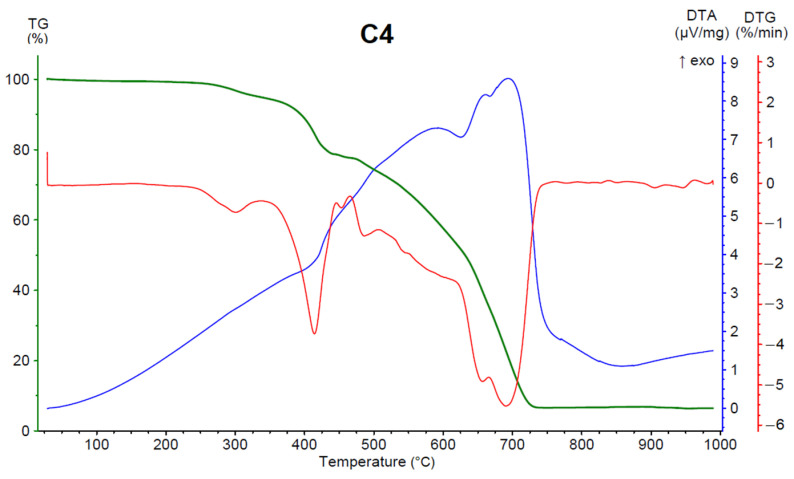
Thermal analyses of zinc (II) coordination compounds. TG (green), DTA (blue) and DTG (red) curves for complexes **C1**–**C4**.

The decomposition pathway of studied compounds (Figure 5, Table 4) is the same as for analogous copper and compounds reported previously [52]. Among studied compounds, only **C1** contains water molecules in the structure. The mass loss corresponding to dehydration indicates one water molecule per one zinc atom (the calculated mass loss is 5.1%). The dehydration starts at a low temperature, which suggests that water molecules are located in the outer coordination sphere. The organic ligand disintegrates in two stages; i.e., the pyridine ring decomposes first, followed by the imidazopyridine part (Table 4). The coordination moieties of **C3** and **C4** start to decompose in lower temperatures in comparison to these of **C1** and **C2**, whose structures were not determined. The organic ligands present in **C1** and **C2** possess nitrogen atoms in positions favoring the formation of polymeric structures. This can be an explanation for the larger thermal stability of **C1** and **C2** since the coordination polymers are usually more temperature-resistant than discrete coordination compounds [53]. A noticeable difference in thermal stability exists also between dinuclear compounds **C3** and **C4**. In this case, the reason lies in their different supramolecular structures. The temperature initiating decomposition is higher for **C3**, which indicates that the sum of intermolecular interaction energies is larger than that in **C4**. The final product after the decomposition of studied compounds is zinc oxide. Its amount is lower than expected (the calculated residual mass is in the range of 19.8–24.5%), most likely due to evaporation of zinc in the form of ZnCl_2_ (m.p. 290 °C [54]) and also possibly of ZnBr_2_ (m.p. 394 °C [54]) in the case of **C4**. The partial removal of zinc content, when the chlorides and bromides are present in the structure, is a known phenomenon [55,56].

### 2.6. Structural Analysis

**Figure 6:**
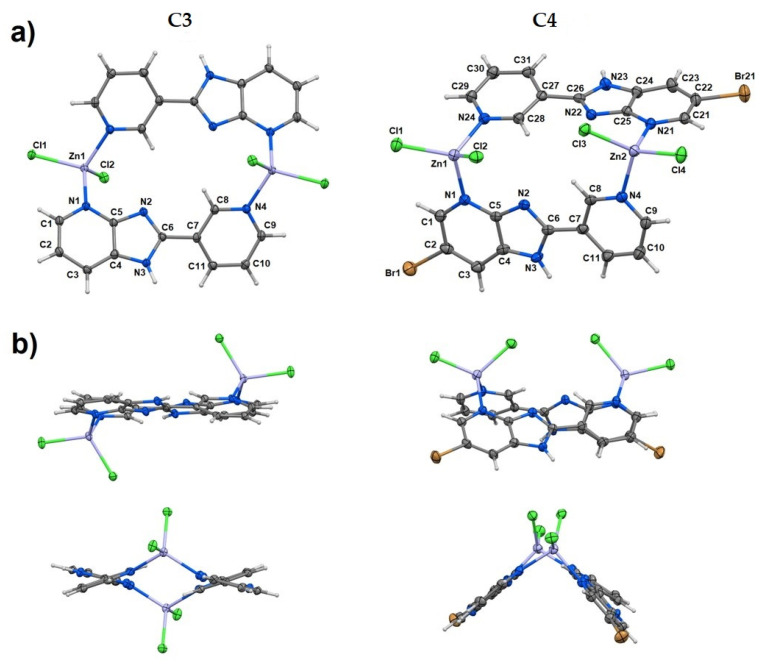
(**a**,**b**) Molecular structures of **C3** and **C4** with displacement ellipsoids of nonhydrogen atoms plotted with 50% probability (**a**). A comparison of the coordination entity structures (**b**).

The diffraction measurement allowed finding the structures of **C3** and **C4**. These are dinuclear coordination compounds of the general formula [Zn_2_Cl_4_(L)_2_]. Zinc cations are connected by two molecules of organic ligand, which are coordinated by nitrogens of both pyridine rings (Figure 6a). The coordination sphere of each central atom is completed by two chlorides; thus, the coordination number is 4. The whole coordination entity of **C4** is symmetry-independent, whereas the asymmetric unit of **C3** contains half of the dinuclear molecule due to an inversion center between zinc cations (special position *h* of the *P*-1 space group). The geometry index for four-coordinated atoms *τ*_4_ is 0.93 for **C3**, and 0.93 and 0.92 for **C4**, which means that all coordination polyhedra possess tetrahedral geometry [57]. The angles of coordination polyhedra are in the range of 96.4–116.7°, which indicates small deviations from ideal tetrahedral parameters (Table 5). Despite the same formula and formed bonds, the structures of coordination entities are different between **C3** and **C4** (Figure 6b). In **C3**, organic ligands are parallel, and the ZnCl_2_ moieties are oppositely oriented, while in **C4**, organic ligands are perpendicular (the dihedral angle between planes defined by imidazopyridine parts of ligand molecules is 87.4°) and the ZnCl_2_ moieties are oriented in the same direction.

The crystal structures of **C3** and **C4** are stabilized by various intermolecular interactions, among which π–π stacking and hydrogen bonds play a crucial role. Due to different structures of coordination entities, the motifs of π–π interactions are also varied (Table 6). In **C3**, non-conjugated pyridine rings, as well as imidazopyridine parts, interact only between themselves, whereas in **C4**, the mixed association is formed. The strongest hydrogen bonds existing in the crystal structure of both studied compounds are N–H•••Cl (Table 7). They form chain motifs, which are propagated along [0 1 0] (in **C4**) and [0 0 1] (in both) crystallographic axes. Besides the above-mentioned interactions, the Cl•••Cl halogen bonds (in **C4**) and the anion–π interactions (Cl•••π in both and Br•••π in **C4**) can also be identified.

In the Cambridge Structural Database, there are no deposited structures of the coordination compounds with ligands L1–L4 [58]. The most similar structurally characterized dinuclear zinc compound is tetrachlorobis{µ-[2-(pyridin-4-yl)-1H-benzimidazole]}dizinc (II), which was obtained in the solvent-free form [59] and as dimethyl sulfoxide solvate [60]. The pyridinyl-benzimidazole molecules form bridges via nitrogen atoms of pyridine and imidazole rings. The ZnCl_2_ moieties are oppositely oriented, similarly to the case of **C3**. The second and the last known hybrid (organic–inorganic) coordination compound consisting of zinc and 2-(pyridin-4-yl)-1H-benzimidazole is diaquabis(nitrato-O)bis[2-(4-pyridyl)-1H-benzimidazole-N]zinc (II) [61]. This is a mononuclear compound due to the monodentate coordination mode of pyridinyl-benzimidazole molecules via a nitrogen of the pyridine ring. The more numerous group of zinc and pyridinyl-benzimidazole compounds also contains organic aromatic co-ligands with two or three carboxylate groups [62,63,64,65,66,67,68,69,70]. Such co-ligands form double or triple bridges, and depending on the coordination mode of pyridinyl-benzimidazole (monodentate or bidentate bridging as described above, or tridentate bridging after deprotonation of imidazole nitrogen) the one-, two- or three-dimensional polymeric networks are propagated.

### 2.7. ADME Analysis

**Figure 7:**
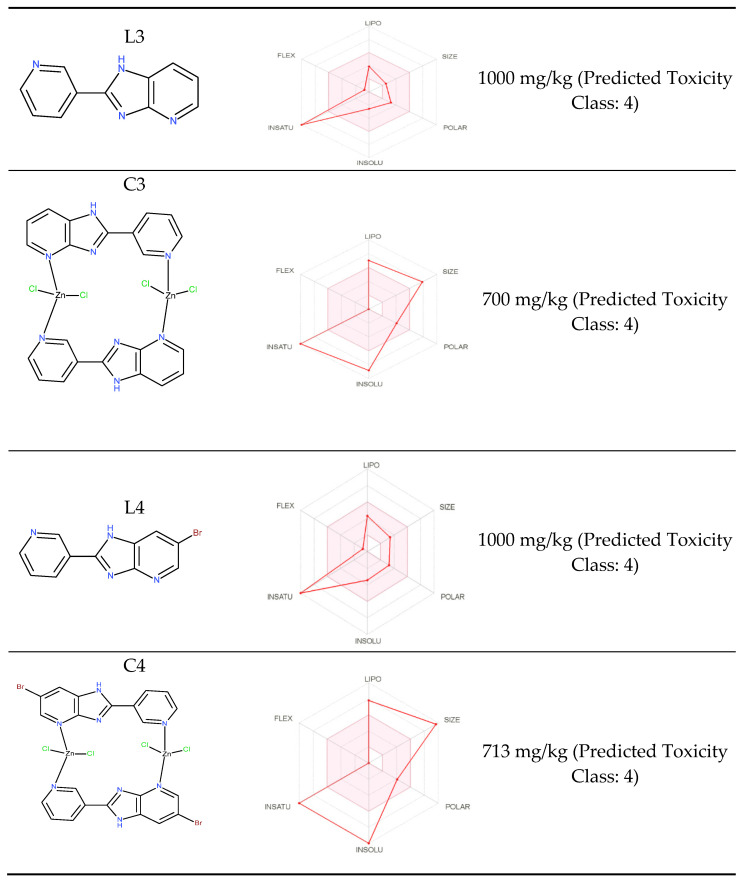
Bioavailability radars for complexes.

The compounds should meet the basic properties that will suggest their usefulness. All tested compounds fulfill the rules of Lipinski [71] and Veber [72]. Bioavailability radars (Figure 7) present six physicochemical properties: lipophilicity, size, polarity, solubility, flexibility and saturation. All compounds have a good bioavailability score of 0.55, calculated relying on the total charge, TPSA and violation of the Lipinski filter. The ligands are absorbed from the gastrointestinal tract and pass the blood–brain barrier. All compounds belong to the fourth class of toxicity (Figure 7). The activity was predicted for the obtained ligands (Table 8). ADME analysis, the possibility of passing the blood–brain barrier and cancer cell line prediction results highlighted the possibility of activity against oligodendroglioma.

## 3. Materials and Methods

### 3.1. Materials and Analysis

Substrates used for ligand synthesis as well as ZnCl_2_·2H_2_O were purchased from Sigma Aldrich (Warszawa, Poland). Glioblastoma (T98G), neuroblastoma (SK-N-AS) and lung adenocarcinoma (A549) cell lines and human normal skin fibroblasts (CCD-1059Sk) were obtained from the American Type Culture Collection (Manassas, VA, USA). DMEM, high glucose; DMEM/F12; MEM; fetal bovine serum; penicillin; and streptomycin were all obtained from Sigma-Aldrich.

### 3.2. Methods and Instruments

Samples of complexes (about 20 mg) were digested in a mixture of concentrated 36% HCl (1 mL) and 65% HNO_3_ (6 mL). The contents of Zn (II) in solid complexes were determined using an F-AAS spectrometer (Analityk Jena, contraAA 300, Jena, Germany) with a continuum source of light and using air/acetylene flame (Analityk Jena, contraAA 300). Absorbances were measured at the analytical spectral line of 213.9 nm for Zn (II). The limit of quantification was 0.004 mg/L for Zn (II). Solid samples were decomposed using the Anton Paar Multiwave 3000 (Graz, Austria) closed-system instrument. Mineralization was carried out for 45 min at 240 °C under the pressure of 60 bar. The contents of carbon, hydrogen and nitrogen were determined with a Vario Micro (Elementar Analysensysteme GmbH, Langenselbold, Germany). FTIR spectra were recorded with an IR Tracer-100 Shimadzu Spectrometer (4000–600 cm^−1^ with an accuracy of recording of 1 cm^−1^, Kyoto, Japan) using KBr pellets. The thermal analyses were carried out with a Netzsch STA 449 F1 Jupiter thermoanalyzer (Netzsch-Geratebau GmbH, Selb, Germany) coupled with Netzsch Aeolos Quadro QMS 403 mass spectrometer (Netzsch-Geratebau GmbH, Selb, Germany). Samples were heated in corundum crucibles up to 1000 °C, with 10 °C/min heating rate, in the atmosphere of synthetic air (20% O_2_, 80% N_2_). The suitable crystals of **C3** and **C4** were selected, and X-ray diffraction data for them were collected on an XtaLAB Synergy Dualflex Pilatus 300K diffractometer (Rigaku Corporation, Tokyo, Japan). Using Olex2 [73], the structures were solved with the SHELXT [74] using intrinsic phasing and refined with SHELXL [75] using least-squares minimization. The details concerning X-ray diffraction data and structure refinement are given in Table 9.

The cytotoxic effect of the compounds was evaluated using glioblastoma (T98G), neuroblastoma (SK-N-AS) and lung adenocarcinoma (A549) cell lines and human normal skin fibroblasts (CCD-1059Sk). T98G and SK-N-AS cells were cultured in DMEM, high glucose; A549 cells were cultured in DMEM/F12; and CCD-1059Sk cells were cultured in MEM. All media were supplemented with 10% FBS, 100 U/mL of penicillin and 100 mg/mL of streptomycin (PenStrep). Cells were incubated at 37 °C in a humidified atmosphere of 5% CO_2_. Stock solutions were prepared by dissolving the compounds in sterile dimethyl sulfoxide (DMSO) to obtain the concentration of 50 mg/mL. The cells were seeded into 96-well sterile plates (Nunc) at a density of 1 × 10^5^ cells/mL. After 24 h of incubation, the medium was removed from each well, and then cells were incubated for the next 24 h with different concentrations of the tested compounds (1–100 µg/mL) in respective medium containing 2% FBS. Cytotoxicity of the compounds was evaluated using the MTT assay [76], which is based on the conversion of 3-(4,5-dimethylthiazol-2-yl)-2,5-diphenyltetrazolium bromide (MTT) into dark-blue formazan crystals. Briefly, after 24 h incubation of cells with varying concentrations of the tested compounds, all culture media were removed from the plates. Cells were washed with PBS, and then 100 µL of medium containing 10% MTT solution (5 mg/mL) was added to each well. After 3 h incubation, 100 µL (per well) of 10% SDS buffer solution was added to solubilize formazan crystals. After overnight incubation, the absorbance was measured at 570 nm using a microplate reader (Epoch, BioTek Instruments). Experiments were repeated twice, and the measurements in each experiment were run in triplicate. The viability of the investigated cells was expressed as a percentage of the viability of the untreated cells, and the results were transformed into IC50 values (expressed as mean ± SD). Zhang F. et al. [77] indicated that the median inhibitory concentration (IC50) of etoposide incubated for 24 h with lung adenocarcinoma (A549) cells was 115 μg/mL [77]. This result is in line with the one obtained in our experiments and presented in Table 2 (IC50 > 100 µg/mL). The complexes were screened for antibacterial and antifungal activities by micro-dilution broth method using Mueller–Hinton broth for growth of bacteria or Mueller–Hinton broth with 2% glucose for growth of fungi [78]. Minimal inhibitory concentrations (MICs) of the tested derivatives were evaluated for the panel of the reference microorganisms from the American Type Culture Collection (ATCC), including Gram-negative bacteria (*Escherichia coli* ATCC 25922, *Salmonella* Typhimurium ATCC14028, *Klebsiella pneumoniae* ATCC 13883, *Pseudomonas aeruginosa* ATCC 9027, *Proteus mirabilis* ATCC 12453), Gram-positive bacteria (*Staphylococcus aureus* ATCC 25923, *Staphylococcus epidermidis* ATCC 12228, *Micrococcus luteus* ATCC 10240, *Enterococcus faecalis* ATCC 29212, *Bacillus subtilis* ATCC 6633, *Bacillus cereus* ATCC 10876) and fungi (*Candida albicans* ATCC 10231, *Candida parapsilosis* ATCC 22019, *C. glabrata* ATCC 90030).

### 3.3. Statistical Analysis

The data were expressed as mean ± SD values from three independent replicate experiments. A *p* value of less than 0.05 compared with the control group was considered to be statistically significant, using Student’s *t*-test analysis [79]

### 3.4. ADME Analysis

An ADME analysis was performed using SwissADME service (Swiss Institute of Bioinformatics 2021) [80,81,82] and ProTOX II service for the prediction of toxicities for tested compounds [83].

Ligands were analyzed using www.way2drug.com/Cell-line (accesse on 10 February 2022) service, a freely available web service for in silico prediction of human cell line cytotoxicity for drug-like compounds [84]. Cell line cytotoxicity predictor (CLC-Pred) is a service for the prediction of the cytotoxic effect of chemical compounds) [85].

## 4. Conclusions

Four new solid-state complexes of Zn (II) with benzimidazole derivatives were synthesized. Only compound **C1** is hydrated. Changes observed in FTIR spectra of complexes indicate that free ligands coordinate to metal (II) ions via N donor atoms from pyridine rings. All compounds are stable at room temperature. The shape of the TG curve, solid and volatile intermediates and final products clearly indicate the obtainment of the coordination compound. The diffraction measurement allowed finding the structures of **C3** and **C4**. These are dinuclear coordination compounds of the general formula [Zn_2_Cl_4_(L)_2_]. Zinc cations are connected by two molecules of organic ligand, which are coordinated by nitrogens of both pyridine rings. The coordination sphere of each central atom is completed by two chlorides; thus, the coordination number is 4. The antimicrobial activity of Zn complexes, free ligands and reference drugs showed no bioactivity against Gram-negative bacteria and yeasts. Regarding Gram-positive bacteria, mild bioactivity was detected, with a slight increase for complexes. All compounds have a good bioavailability score of 0.55. The ligands are absorbed from the gastrointestinal tract and pass the blood–brain barrier. All compounds belong to the fourth class of toxicity. ADME analysis, the possibility of passing the blood–brain barrier and cancer cell line prediction results highlighted the possibility of activity against oligodendroglioma. The MTT test confirmed that the conversion of free ligands into coordination compounds significantly increased their anticancer activity against glioblastoma, neuroblastoma and lung carcinoma cell lines. The synthesized compounds are stronger than etoposide, which is a component of the treatment of glioblastoma, neuroblastoma and lung cancer. Due to cytotoxicity in relation to normal cells, a different method of drug delivery should be considered than classical chemotherapy. Treatment of neoplastic cells directly into the exposed place in the brain with a cytostatic drug or encapsulation of the chemotherapeutic agent could minimize the risk of damage to healthy cells. Comparing the coordination compounds of copper (II) [52] and zinc (II), we notice a strong influence of the metal ion on the anticancer properties of the studied coordination compounds. The electronic structure of the metal ion, its biological activity and the type of bond formed between the metal ion and the organic ligand play a significant role in the biological activity of the studied complexes.

## Figures and Tables

**Table 1:** Percentage composition of the elements in coordination compounds.

C1 [Zn_2_Cl_4_ (L1)_2_]∙2H_2_O (C_22_H_20_N_8_O_2_Zn_2_Cl_4_)	C2 [Zn_2_Cl_4_ (L2)_2_] (C_22_H_16_N_8_Zn_2_Cl_4_)	C3 [Zn_2_Cl_4_ (L3)_2_] (C_22_H_14_N_8_Zn_2_Cl_4_)	C4 [Zn_2_Cl_4_ (L4)_2_] (C_22_H_14_N_8_Zn_2_Cl_4_Br_2_)
M = 701.5093 g/mol	M = 664.4940 g/mol	M = 662.9722 g/mol	M = 822.7802 g/mol
yield (43%)	yield (38%)	yield (41%)	yield (38%)
calculated/found (%)	calculated/found (%)	calculated/found (%)	calculated/found (%)
C 37.69/37.75	C 39.73/39.81	C 39.86/39.93	C 32.11/32.17
H 2.87/2.90	H 2.42/2.47	H 2.13/2.15	H 1.71/1.76
N 15.98/16.00	N 16.85/16.68	N 16.90/16.75	N 13.62/13.54
O 4.56/4.45	-	-	-
Zn 18.65/18.75	Zn 19.66/19.69	Zn 19.72/19.82	Zn 15.89/15.67
Cl 20.23/20.15	Cl 21.32/21.35	Cl 21.39/21.35	Cl 17.23/17.33
-	-	-	Br 19.42/19.53

**Table 2:** Cytotoxic effect of the investigated compounds against glioblastoma (T98G), neuroblastoma (SK-N-AS), lung adenocarcinoma (A549) cell lines and human normal fibroblasts (CCD-1059Sk) determined by MTT assay after 24 h incubation.

IC_50_ ± SD (µg/mL)				
Complex	T98G	SK-N-AS	A549	CCD-1059-Sk
**L1**	41.25 ± 2.30	>100	>100	>100
**C1**	32.22 ± 0.92	35.59 ± 1.03	33.51 ± 1.29	18.42 ± 0.37
**L2**	34.98 ± 1.44	81.35 ± 3.31	43.08 ± 2.17	>100
**C2**	24.29 ± 0.11	33.72 ± 0.39	34.44 ± 0.75	27.27 ± 1.05
**L3**	>100	>100	>100	>100
**C3**	46.54 ± 1.86	41.60 ± 1.93	41.34 ± 2.17	30.84 ± 1.11
**L4**	>100	>100	>100	>100
**C4**	30.05 ± 1.81	36.17 ± 0.44	35.01 ± 0.86	33.62 ±0.85
**Etoposide**	>100	67.83 ± 2.03	>100	>100

**Table 3:** In vitro antimicrobial activity of ligands and complexes tested.

Chemicals Microorganism	L1	C1	L2	C2	L3	C3	L4	C4	Ref Drugs
	MIC (mg/L)								
**Gram-Positive Bacteria**									**Van**
*S. aureus* ATCC 25923	>1000	1000	1000	500	>1000	1000	>1000	>1000	0.98
*S. epidermidis* ATCC 12228	>1000	500	1000	500	>1000	1000	1000	500	0.98
*M. luteus* ATCC 10240	500	250	250	250	500	500	>1000	500	0.12
*E. faecalis* ATCC 29212	>1000	>1000	1000	1000	>1000	>1000	1000	>1000	1.95
*B. subtilis* ATCC 6633	>1000	1000	500	500	1000	>1000	1000	>1000	0.24
*B. cereus* ATCC 10876	>1000	>1000	250	500	1000	>1000	1000	>1000	0.98
**Gram-Negative Bacteria**									**Cip**
*S. typhimurium* ATCC 14028	>1000	>1000	1000	1000	>1000	>1000	>1000	>1000	0.061
*E. coli* ATCC 25922	>1000	>1000	1000	1000	>1000	>1000	>1000	>1000	0.015
*P. mirabilis* ATCC 12453	>1000	1000	1000	1000	>1000	>1000	>1000	>1000	0.03
*K. pneumoniae* ATCC 13883	>1000	>1000	>1000	1000	>1000	>1000	>1000	>1000	0.12
*P. aeruginosa* ATCC 9027	>1000	>1000	>1000	>1000	>1000	>1000	>1000	>1000	0.49
**Yeasts**									**Nys**
*C. glabrata* ATCC 2091	>1000	>1000	>1000	>1000	>1000	>1000	>1000	>1000	0.48
*C. albicans* ATCC 102231	>1000	>1000	1000	1000	>1000	>1000	500	>1000	0.24
*C. parapsilosis* ATCC 22019	>1000	>1000	>1000	>1000	1000	>1000	1000	>1000	0.24

**Table 4:** Temperature ranges and mass losses of thermal decomposition stages of cadmium (II) coordination compounds.

	C1	C2	C3	C4	m/z
Dehydration	50–120 °C 5.5%	-	-	-	17, 18
1st stage of ligand decomposition	350–540 °C 17.8%	360–520 °C 20.5%	310–450 °C 25.2%	260–450 °C 21.5%	12, 17, 18, 26, 27, 29, 30, 44, 45, 50, 52
2nd stage of ligand decomposition	540–725 °C 64.1%	520–760 °C 75.9%	450–710 °C 57.2%	450–740 °C 72.2%	12, 13, 17, 18, 22, 26, 27, 29, 30, 35, 36, 37, 38, 41, 44, 45, 46, 70, 72, 79 *, 81 *, 158 *, 160 *, 162 *
Residual mass	12.6%	11.7%	17.6%	6.3%	-

* signals present only in the mass spectra of **C4**.

**Table 5:** Structural data of the coordination polyhedra in **C3** and **C4**.

i–j	d_ij_ (Å)	i–j–k	α_ijk_ (°)
**C3**			
Zn1–Cl1	2.2667(5)	Cl1–Zn1–Cl2	113.01(2)
Zn1–Cl2	2.2272(5)	Cl1–Zn1–N1	103.72(5)
Zn1–N1	2.0235(15)	Cl1–Zn1–N4(i)	108.43(5)
Zn1–N4(i)	2.0391(15)	Cl2–Zn1–N1	114.30(5)
		Cl2–Zn1–N4(i)	102.87(5)
		N1–Zn1–N4(i)	114.68(6)
**C4**			
Zn1–Cl1	2.2115(9)	Cl1–Zn1–Cl2	113.26(3)
Zn1–Cl2	2.2295(9)	Cl1–Zn1–N1	109.05(7)
Zn1–N1	2.077(3)	Cl1–Zn1–N24	115.19(8)
Zn1–N24	2.030(3)	Cl2–Zn1–N1	113.71(8)
		Cl2–Zn1–N24	103.92(8)
		N1–Zn1–N24	101.15(10)
Zn2–Cl3	2.2304(9)	Cl3–Zn2–Cl4	116.72(3)
Zn2–Cl4	2.2261(9)	Cl3–Zn2–N21	114.10(8)
Zn2–N21	2.079(3)	Cl3–Zn2–N4	107.83(8)
Zn2–N4	2.048(3)	Cl4–Zn2–N21	106.33(7)
		Cl4–Zn2–N4	113.75(8)
		N21–Zn2–N4	96.4(1)

Symmetry transformations used to generate equivalent atoms: (i) −*x* + 1, −*y* + 1, −*z* + 1.

**Table 6:** Stacking interactions in the structures of **C3** and **C4**. Each ring is indicated by one atom, which belongs solely to this ring. The α is a dihedral angle between planes I and J, β is an angle between Cg(I)–Cg(J) vector and normal to plane I, d_p_ is a perpendicular distance of Cg(I) on ring J plane.

R(I)•••R(J)	d(Cg•••Cg) (Å)	α (°)	β (°)	d_p_ (Å)
**C3**				
N2•••N1(i)	4.2575(10)	1.44(9)	29.9	3.7399(7)
N1•••N1(ii)	3.6918(9)	0.03(8)	23.5	3.3855(7)
N4•••N4(iii)	3.7548(10)	0.00(8)	26.8	3.3517(7)
**C4**				
N1•••N2(iv)	4.0721(18)	0.72(16)	25.0	3.6686(12)
N1•••N1(iv)	3.8205(16)	0.03(14)	15.2	3.6865(12)
N1•••N24(v)	3.8155(16)	10.00(14)	24.1	3.4508(12)
N2•••N22(v)	3.4906(17)	5.88(17)	9.3	3.3863(13)
N4•••N21(v)	3.6557(16)	5.72(14)	21.2	3.3775(12)

Symmetry transformations used to generate equivalent atoms: (i) −*x* + 2, −*y* + 1, −*z* + 1; (ii) −*x* + 2, −*y* + 2, −*z* + 1; (iii) −*x* + 1, −*y* + 1, −*z*; (iv) −*x* + 1, −*y* + 1, −*z* + 1; (v) *x*, −*y* + 0.5, *z* – 0.5; (vi) −*x* + 2, −*y* + 1, −*z* + 2.

**Table 7:** Hydrogen bonds in the structures of **C3** and **C4**.

D–H•••A	d(D–H) (Å)	d(H•••A) (Å)	d(D•••A) (Å)	<(DHA) (°)	Graph-Set
**C3**					
N3–H3•••Cl1(i)	0.88	2.39	3.2688(16)	173	C (7)
**C4**					
N3–H3•••Cl3(ii)	0.88	2.57	3.195(3)	129	C (8)
N3–H3•••Cl4(iii)	0.88	3.02	3.722(3)	139	C (8)
N23–H23•••Cl2(iv)	0.88	2.71	3.459(3)	143	C (8)
N23–H23•••Cl2(v)	0.88	2.64	3.237(3)	126	C (8)

Symmetry transformations used to generate equivalent atoms: (i) *x*, *y*, *z* − 1; (ii) *x*, −*y* + 0.5, *z* – 0.5; (iii) −*x* + 2, *y* + 0.5, –*z* + 1.5; (iv) −*x* + 1, *y* + 0.5, −*z* + 1.5; (v) *x*, −*y* + 0.5, *z* + 0.5.

**Table 8:** Cancer cell line prediction results for the ligand—Pa (probability “to be active”), Pi (probability “to be inactive”).

Ligand	Pa	Pi	Cell-Line Name	Tissue	Tumor Type
L3	0.587	0.029	Oligodendroglioma	Brain	Glioma
L3	0.538	0.010	Colon adenocarcinoma	Colon	Adenocarcinoma
L3	0.490	0.022	Non-small-cell lung carcinoma	Lung	Carcinoma
L3	0.475	0.009	Pancreatic carcinoma	Pancreas	Carcinoma
L3	0.439	0.043	Pancreatic carcinoma	Pancreas	Carcinoma
L4	0.559	0.006	Pancreatic carcinoma	Pancreas	Carcinoma
L4	0.554	0.009	Colon adenocarcinoma	Colon	Adenocarcinoma
L4	0.415	0.038	Cervical adenocarcinoma	Cervix	Adenocarcinoma
L4	0.426	0.099	Oligodendroglioma	Brain	Glioma

**Table 9:** X-ray diffraction data and structure refinement details for **C3** and **C4**.

Compound	C3	C4
Empirical formula	C_22_H_16_Cl_4_N_8_Zn_2_	C_22_H_14_Br_2_Cl_4_N_8_Zn_2_
Formula weight	664.97	822.77
Temperature (K)	99.97(4)	101(2)
Crystal system	Triclinic	Monoclinic
Space group	*P*-1	*P*2_1_/*c*
*a* (Å)	7.4842(2)	12.9125(2)
*b* (Å)	8.1366(2)	13.8888(1)
*c* (Å)	9.9889(2)	15.3249(2)
*α* (°)	84.703(2)	90
*β* (°)	87.371(2)	105.483(1)
*γ* (°)	80.218(2)	90
Volume (Å^3^)	596.60(3)	2648.61(6)
*Z*	1	4
Calculated density (g/cm^3^)	1.851	2.063
Absorption coefficient (mm^−1^)	6.858	9.743
*F(000)*	332	1600
Crystal size (mm)	0.063 × 0.041 × 0.026	0.160 × 0.018 × 0.015
Radiation	Cu *Kα* (λ = 1.54184 Å)	Cu *Kα* (λ = 1.54184 Å)
*θ* range for data collection (°)	4.447 to 78.664	3.552 to 78.928
Index ranges	−9 ≤ h ≤ 9 −9 ≤ k ≤ 10 −12 ≤ l ≤ 11	−16 ≤ h ≤ 14 −17 ≤ k ≤ 13 −18 ≤ l ≤ 18
Reflections collected/independent	11156/2407	27186/5268
R*_int_*	0.0386	0.0393
Completeness (%)	99.9	99.9
Data/restraints/parameters	2407/0/163	5268/0/343
Goodness of fit on *F*^2^	1.073	1.026
Final *R* indices (*I* > 2σ(*I*))	*R*1 = 0.0251, *wR*2 = 0.0717	*R*1 = 0.0310, *wR*2 = 0.0815
Final *R* indices (all data)	*R*1 = 0.0267, *wR*2 = 0.0726	*R*1 = 0.0374, *wR*2 = 0.0848
Largest diff. peak/hole (e•Å^−3^)	0.53/−0.34	1.07 and −0.91

## Data Availability

Not applicable.
